# Evaluation of Posttraumatic Headache Phenotype and Recovery Time After Youth Concussion

**DOI:** 10.1001/jamanetworkopen.2021.1312

**Published:** 2021-03-08

**Authors:** Joshua Kamins, Rachel Richards, Bradley J. Barney, Christopher Locandro, Christina F. Pacchia, Andrew C. Charles, Lawrence J. Cook, Gerard Gioia, Christopher C. Giza, Heidi K. Blume

**Affiliations:** 1 Goldberg Migraine Program, Department of Neurology, David Geffen School of Medicine at University of California, Los Angeles; 2 UCLA Steve Tisch BrainSPORT Program, Department of Neurosurgery, David Geffen School of Medicine at University of California, Los Angeles; 3 Department of Pediatrics, University of Utah, Salt Lake City; 4 Division of Pediatric Neuropsychology, Children's National Hospital, SCORE Program, Rockville, Maryland; 5 Department of Pediatrics, Pediatric Neurology Division, UCLA Mattel Children’s Hospital, University of California, Los Angeles; 6 Division of Pediatric Neurology, Seattle Children’s Hospital, University of Washington, Seattle

## Abstract

**Importance:**

The Four Corners Youth Consortium was created to fill the gap in our understanding of youth concussion. This study is the first analysis of posttraumatic headache (PTH) phenotype and prognosis in this cohort of concussed youth.

**Objective:**

To describe the characteristics of youth with PTH and determine whether the PTH phenotype is associated with outcome.

**Design, Setting, and Participants:**

This cohort study examined outcomes from patients in a multi-institutional registry of traumatic brain injury (TBI) clinics from December 2017 to June 2019. Inclusion criteria included being between ages 5 and 18 years at enrollment and presentation within 8 weeks of a mild TBI. Data were analyzed between February 2019 and January 2021.

**Exposure:**

Mild TBI with standard care.

**Main Outcomes and Measures:**

Time to recovery and headache 3 months after injury; measurement device is the Postconcussion Symptom Inventory (PCSI). PTH with migraine phenotype was defined as moderate-severe headache that is new or significantly worse compared with baseline and associated with nausea and/or photophobia and phonophobia.

**Results:**

A total of 612 patients with 625 concussions were enrolled, of whom 387 patients with 395 concussions consented to participate in this study. One hundred nine concussions were excluded (concussions, rather than patients, were the unit of analysis), leaving 281 participants with 286 concussions (168 [58.7%] girls; 195 [75.6%] White; 238 [83.2%] aged 13-18 years). At the initial visit, 133 concussions (46.5%) were from patients experiencing PTH with a migraine phenotype, 57 (20%) were from patients experiencing PTH with a nonmigraine phenotype, and 96 (34%) were from patients with no PTH. Patients with any PTH after concussion were more likely to have prolonged recovery than those without PTH (median [interquartile range], 89 [48-165] days vs 44 [26-96] days; log-rank *P* < .001). Patients with PTH and a migraine phenotype took significantly longer to recover than those with nonmigraine phenotype (median [interquartile range], 95 [54-195] days vs 70 [46-119] days; log-rank *P* = .01). Within each phenotype, there was no significant difference between sexes in recovery or PTH at 3 months.

**Conclusions and Relevance:**

PTH with a migraine phenotype is associated with persistent symptoms following concussion compared with nonmigraine PTH or no PTH. Given that female sex is associated with higher rates of migraine and migraine PTH, our finding may be one explanation for findings in prior studies that girls are at higher risk for persistent postconcussion symptoms than boys.

## Key Points

**Question:**

Are outcomes for posttraumatic headache (PTH) after youth concussion associated with migraine phenotype?

**Findings:**

In this cohort study from the Four Corners Youth Consortium including 281 patients with 286 concussions, PTH with migraine phenotype was associated with prolonged recovery and higher risk of persistent PTH following concussion compared with nonmigraine PTH phenotype or no PTH.

**Meaning:**

These findings suggest that PTH with migraine phenotype may be a marker for more significant injury or deleterious pathophysiology after head injury and could be a target for early intervention to prevent persistent and disabling symptoms following concussion.

## Teaser

This cohort study of youth experiencing posttraumatic headache following concussion examines the association between migraine phenotype and recovery outcomes.

## Introduction

Concussions and mild traumatic brain injuries (mTBI) are common among children and adolescents and constitute a major public health challenge. While symptoms from concussion typically resolve days to weeks after injury, 10% to 30% of patients have symptoms that last longer than 4 weeks, and a smaller proportion have symptoms that persist for far longer.^1,2,3^ Evidence suggests adolescents are at particularly high risk in terms of symptom burden and time to recovery.^1,4^

Posttraumatic headache (PTH), defined by the *International Classification of Headache Disorders (Third Edition)* as a new or significantly worsened head pain attributed to a head injury,^5^ is the most common acute and persistent symptom of concussion.^3,6^ Furthermore, PTH with migraine features may be an early indication of prolonged symptoms after concussion.^7^ Although adolescents have a higher risk for sustaining concussions and developing persistent symptoms than younger children or adults, there is little data regarding PTH recovery and treatment in youth. A 2013 report by the Institute of Medicine^8^ highlighted the knowledge gap concerning youth concussion and recovery. Researchers have identified risk factors for prolonged recovery in youth including female sex, prior headache history, and a high number of acute symptoms; however, there is much that remains unknown.^1,2,8^ Additionally, as the Childhood and Adolescent Migraine Prevention trial^9^ showed, the treatment of pediatric headache is not as simple as applying our knowledge of adult headaches to children. There are unique factors involved in the management of childhood headache that merit special attention, including differences in migraine symptoms and response to treatment.^10,11^

The Four Corners Youth Consortium (4CYC) was created to help fill the gaps in our understanding of youth concussion and recovery. The consortium has undertaken a multicenter, prospective, observational cohort study of youth concussion. 4CYC collects comprehensive data, including demographic and injury characteristics, clinical and treatment variables, and outcomes. This database presents a unique opportunity to examine PTH phenotypes and treatment following youth concussion, as well as to analyze the relationships between PTH phenotype, patient and injury characteristics, and recovery. We hypothesized that female sex and PTH with a migraine phenotype would be associated with prolonged recovery from concussion and persistent PTH.

The primary objective of this study was to determine if PTH phenotype following pediatric concussion (mTBI) was associated with a longer time to resolution of postconcussion symptoms or with the risk of persistent PTH 3 and 6 months after injury. We also describe treatments used to manage PTH and compare baseline characteristics between youth with migraine and nonmigraine PTH phenotypes after concussion.

## Methods

### Data Source: 4CYC Registry

As outlined in a recent publication,^12^ the 4CYC registry is a multi-institutional collaboration between 3 academic centers with expertise in youth concussion care and research: the Safe Concussion Outcome, Recovery, and Education (SCORE) program at Children’s National Hospital; Seattle Children’s Hospital and University of Washington Medical Center; and the UCLA Steve Tisch BrainSPORT program at UCLA Mattel Children’s Hospital. The 4CYC is supported by the University of Utah Data Coordinating Center. Throughout this study, the single institutional review board (sIRB) model^13^ has been used, with the University of Utah’s IRB serving as the sIRB. This study followed the Strengthening the Reporting of Observational Studies in Epidemiology (STROBE) reporting guideline for cohort studies.

### Enrollment and Consent

Eligible patients in the registry were between ages 5 to 18 years at enrollment and presented to a participating specialty mTBI clinic with a referral diagnosis of mTBI or concussion within 8 weeks of injury. After referral, diagnosis of mTBI was confirmed by clinicians with specialized expertise in TBI. A patient may be enrolled in the registry for more than 1 concussion at separate time points (5 of 281 patients [1.8%] with 2 concussions each in this study).

Patients were excluded for a Glasgow Coma Scale (GCS) score of less than 13, a penetrating injury, or an inability to read or sign for consent (and without parental consent). Although the registry also contains routine clinical data on patients enrolled under a waiver of consent, these data are inadequate for ascertaining longitudinal outcomes; therefore, patients who did not consent are excluded from our primary analyses. Informed consent for follow-up and, as applicable, assent were obtained per site-specific IRB regulations.

### Measures

Measures collected in the registry include demographic characteristics, personal and family medical/psychiatric history, injury and treatment details, neurological assessments, and other National Institutes of Health common data elements for pediatric TBI and sports concussions.^14^ A primary measure was the Postconcussion Symptom Inventory (PCSI),^15^ which assesses preinjury and current postinjury symptoms to generate a Retrospective Adjusted Post-Injury Difference (RAPID) score (ie, subtracting preinjury rating from postinjury rating on PCSI) with both parent-reported (for patients aged 8-12 years or with missing adolescent PCSI) and age-appropriate child-reported versions (for patients aged ≥13 years).^16^ Collection of this information is standard care at 4CYC clinics at the initial visit.

### Headache Phenotypes

We characterized each patient’s PTH by applying data from PCSI questionnaires to match validated clinical criteria specified in the *ICHD-3*.^5^ The PCSI asks patients to rate their current and preinjury symptoms on a 7-point dimensional scale with values ranging from 0 to 6. Patients were considered to have PTH if their headache RAPID score at the initial visit was 2 or more (ie, postinjury headache rating was 2 or more points higher than preinjury status on PCSI), a value determined to exceed the 80% confidence interval for change from baseline.^16^ In other words, PTH was defined as the presence of a headache that was rated 2 or more points higher at a patient’s first clinic visit compared with their preinjury rating of headache. If change in headache was less than 2 points higher than baseline at the first visit after injury, the patient was classified as not having PTH. Patients with a preinjury headache score of 5 or greater were excluded, because the upper limit of the PCSI scale is 6 and it would be impossible to determine if the headache was significantly worse after injury. PTH was classified as migraine phenotype (PTH-M) if the intensity was moderate to severe with a score of 3 or greater on the headache question on PCSI at first visit, and if they endorsed a score on nausea of 1 or greater or both photophobia (ie, sensitivity to light ≥1) and phonophobia (sensitivity to noise ≥1) on the PCSI. The PTH was considered to be nonmigraine (PTH-NM) if it did not meet criteria for migraine headache.

### Outcomes

The primary outcomes were time to recovery and concussion-attributable headache 3 months after injury; secondary outcome was headache 6 months after injury. Recovery was defined as resolution of symptoms related to concussion. Among participants with documented recovery, clinician report determined recovery date. If there was no clinician-reported recovery date, the parent-reported recovery date was used. Parent-reported information was collected via survey for patients at 3, 6, 9, and 12 months postinjury until documented recovery. Parents were asked to state if and when the child had recovered on each survey. Once recovery was documented, no further surveys were sent.

Presence of concussion-related headache at 3 and 6 months was determined by report on the corresponding survey. Parents of unrecovered patients were asked, “As a result of the concussion, has your child experienced ANY of these physical symptoms ANY MORE THAN USUAL today or in the past day?” and could select “Headaches.” Patients without follow-up survey data but for whom the documented time to recovery was 104 days or less for the 3-month survey, or 194 days or less for the 6-month survey, were presumed to not have concussion-related headache at the indicated assessment or thereafter.

### Statistical Analysis

Exclusions specific to this study in the 4CYC data set included missing or invalid injury date, invalid recovery date (including recovery date being identical to the injury date), incomplete information on demographic characteristics, incomplete PCSI data, preinjury headache with a PCSI score of 5 or greater, or enrollment under waiver of consent.

Summaries of patient and injury characteristics and treatment information were created by headache phenotype. Fisher exact tests or Kruskal-Wallis tests were used, as appropriate, to test for differences in these measures between PTH-M and PTH-NM. For the primary outcome of time to recovery, Kaplan-Meier curves were constructed to compare headache phenotypes while accounting for censoring. Log-rank tests for differences in recovery curves focused on PTH-M vs PTH-NM and on PTH vs no PTH; a post hoc exploratory analysis simultaneously compared PTH-M, PTH-NM, and no PTH. Headache at 3- and 6-month postinjury compared PTH-M with PTH-NM using a Fisher exact test. Exploratory analyses included tests for differences in primary outcomes between sexes within each headache phenotype, and between descriptive comparisons of treatment recommendations and multivariable outcome models. Because of the possibility that associations between outcomes and headache phenotype are attributable to associations with age or sex, multivariable models were fitted to relate outcomes to headache phenotype (PTH-M vs PTH-NM), age, sex, and a phenotype × sex interaction. Time to recovery was modeled with the Cox proportional hazards model, and posttraumatic headache at 3 months was modeled with logistic regression.

Data were analyzed from February 2019 to January 2021. All analyses were conducted using SAS version 9.4 (SAS Institute Inc) and all hypothesis tests were declared significant at 2-sided *P* < .05.

## Results

### Demographic and Injury Characteristics

Between December 2017 and June 2019, 612 patients with 625 concussions were enrolled in the 4CYC registry, of whom 387 patients with 395 concussions consented to participate in this study. Of the 395, 109 concussion episodes (27.6%) were excluded, leaving 281 participants with 286 concussions (168 [58.7%] girls, 195 [75.6%] White, 238 [83.2%] aged 13-18 years). There were higher proportions of boys, younger children, patients using public insurance, and patients with a family history of attention-deficit/hyperactivity disorder (ADHD) in the excluded group than the study cohort (eFigure, eTable 1 in the Supplement).

At the initial visit, 133 concussions (47%) were recorded from patients in the PTH-M group, 57 (20%) were recorded from patients in the PTH-NM group, and 96 (34%) were from patients with no PTH. Patient and injury characteristics, as well as differences between the PTH-M and PTH-NM populations, are summarized in Table 1 (age and significant differences) and eTable 2 (nonsignificant differences) in the Supplement.

**Table 1.** Significant Patient and Event Characteristics by Headache Status at Initial Clinic Visit

Characteristic	Concussions, No. (%)^a^				*P* value for PTH-M vs PTH-NM
	Headache phenotype at initial visit			Overall (n = 286)	
	PTH-M (n = 133)	PTH-NM (n = 57)	No PTH (n = 96)		
Age at injury, y					
5-12	19/133 (14.3)	7/57 (12.3)	22/96 (22.9)	48/286 (16.8)	.82^b^
13-18	114/133 (85.7)	50/57 (87.7)	74/96 (77.1)	238/286 (83.2)	
Sex					
Male	41/133 (30.8)	27/57 (47.4)	50/96 (52.1)	118/286 (41.3)	.03^b^
Female	92/133 (69.2)	30/57 (52.6)	46/96 (47.9)	168/286 (58.7)	
Insurance type					
Medicaid/state CHIP	17/132 (12.9)	2/57 (3.5)	9/95 (9.5)	28/284 (9.9)	.02^b^
Commercial	114/132 (86.4)	53/57 (93.0)	82/95 (86.3)	249/284 (87.7)	
Medicare	1/132 (0.8)	0/57	0/95	1/284 (0.4)	
No insurance/self-pay	0/132	2/57 (3.5)	4/95 (4.2)	6/284 (2.1)	
Patient medical history					
Depression	18/120 (15.0)	2/52 (3.8)	6/90 (6.7)	26/262 (9.9)	.04^b^
Migraines	30/119 (25.2)	1/51 (2.0)	24/89 (27.0)	55/259 (21.2)	<.001^b^
Family medical history					
ADHD	21/98 (21.4)	2/45 (4.4)	17/80 (21.3)	40/223 (17.9)	.01^b^
Total No. of family comorbidities					
0	33/95 (34.7)	20/44 (45.5)	27/79 (34.2)	80/218 (36.7)	.05^c^
1	22/95 (23.2)	11/44 (25.0)	22/79 (27.8)	55/218 (25.2)	
2	13/95 (13.7)	10/44 (22.7)	16/79 (20.3)	39/218 (17.9)	
≥3	27/95 (28.4)	3/44 (6.8)	14/79 (17.7)	44/218 (20.2)	
Headache PCSI					
At baseline (preinjury)					
0	91/133 (68.4)	56/57 (98.2)	54/96 (56.3)	201/286 (70.3)	<.001^c^
1-2	30/133 (22.6)	1/57 (1.8)	32/96 (33.3)	63/286 (22.0)	
3-4	12/133 (9.0)	0/57	10/96 (10.4)	22/286 (7.7)	
At initial clinic visit					
0	0/133	0/57	43/96 (44.8)	43/286 (15.0)	<.001^c^
1-2	0/133	32/57 (56.1)	43/96 (44.8)	75/286 (26.2)	
3-4	83/133 (62.4)	22/57 (38.6)	9/96 (9.4)	114/286 (39.9)	
5-6	50/133 (37.6)	3/57 (5.3)	1/96 (1.0)	54/286 (18.9)	
Headache RAPID score					
<1	0/133	0/57	61/96 (63.5)	61/286 (21.3)	<.001^c^
1-2	15/133 (11.3)	33/57 (57.9)	35/96 (36.5)	83/286 (29.0)	
3-4	92/133 (69.2)	21/57 (36.8)	0/96	113/286 (39.5)	
5-6	26/133 (19.5)	3/57 (5.3)	0/96	29/286 (10.1)	

Abbreviations: ADHD, attention-deficit/hyperactivity disorder; CHIP, Child Health Insurance Plan; PCSI, Postconcussion Symptom Inventory; PTH, posttraumatic headache; PTH-M, posttraumatic headache with migraine; PTH-NM, posttraumatic headache with nonmigraine; RAPID, Retrospective Adjusted Post-Injury Difference.

^a^ Patients may be enrolled for more than 1 concussion at different time points; data summarized is at the concussion level.

^b^ Determined using a Fisher exact test.

^c^ Determined using a Kruskal-Wallis test.

Characteristics associated with PTH-M phenotype included female sex (92 of 133 concussions [69.2%] vs 30 of 57 [52.6%] in the PTH-NM group; *P* = .03), depression at baseline (eg, 18 of 120 concussions [15.0%] vs 2 of 52 [3.8%] in the PTH-NM group; *P* = .04), history of migraine (eg, 30 of 119 concussions [25.2%] vs 1 of 51 [2.0%] in the PTH-NM group; *P* < .001), public insurance (eg, Medicaid/state CHIP or Medicare, 18 of 132 concussions [13.6%] vs commercial insurance, 2 of 57 [3.5%] in the PTH-NM group; *P* = .02), and family history of ADHD (eg, 21 of 98 concussions [21.4%] vs 2 of 45 [4.4%] in the PTH-NM group; *P* = .01) (Table 1). There was no significant difference in the time between injury and clinic visit between PTH-M and PTH-NM groups (eTable 2 in the Supplement). Headache noted on PCSI prior to injury was more common in the PTH-M group (eg, PCSI score, 1-2: PTH-M, 30 of 133 concussions [22.6%] vs PTH-NM, 1 of 57 concussions [1.8%]; *P* < .001) (Table 1). Injury characteristics were similar between the 2 groups, and most were sports-related (eTable 2 in the Supplement).

### Treatment

At least 89% of patients in both the PTH-M and PTH-NM groups received counseling regarding management of cognitive and physical activity following concussion. Those with PTH-M were more likely to receive counseling regarding cognitive activity (118 of 133 [88.7%] vs 42 of 57 [73.7%] in the PTH-NM group; *P* = .02). Education about headache, sleep hygiene, and emotional response following concussion was provided to over 80% of both groups (eTable 3 in the Supplement).

Abortive medications were recommended for treatment of 62 concussions (47%) for patients in the PTH-M group and 28 concussions (49%) for patients in the PTH-NM group. The following abortive headache medications were recommended: acetaminophen (PTH-M, 53 concussions [40%] vs PTH-NM, 23 [40%]), ibuprofen (PTH-M, 52 concussions [39%] vs PTH-NM, 24 [42%]), naproxen (PTH-M, 12 concussions [9%] vs PTH-NM, 9 [16%]), rizatriptan or sumatriptan (PTH-M, 7 concussions [5%] vs PTH-NM, 1 [2%]). Prescription preventive medications were prescribed for 28 concussions (21%) for patients in the PTH-M group and 5 concussions (9%) for patients in the PTH-NM group (*P* = .04). Amitriptyline or nortriptyline were prescribed for treatment of 16 concussions (12%) for patients in the PTH-M group and 3 concussions (5%) for patients in the PTH-NM group. Topiramate, cyproheptadine, propranolol, gabapentin, memantine, and venlafaxine were each prescribed to 3% or less of patients with PTH-M and no youth in the PTH-NM group. Nonprescription treatments for headache prevention were recommended for treatment of 53 concussions (40%) for patients with PTH-M and 21 concussions (37%) for patients in the PTH-NM group; specific treatments included melatonin (PTH-M, 41 concussions [31%] vs PTH-NM, 14 [25%]), magnesium (PTH-M, 36 concussions [27%] vs PTH-NM, 15 [26%]), riboflavin (PTH-M, 33 concussions [25%] vs PTH-NM, 13 [23%]), butterbur (PTH-M, 11 concussions [8%] vs PTH-NM, 5 [9%]) (eTable 4 in the Supplement).

### Outcomes

**Figure 1.**
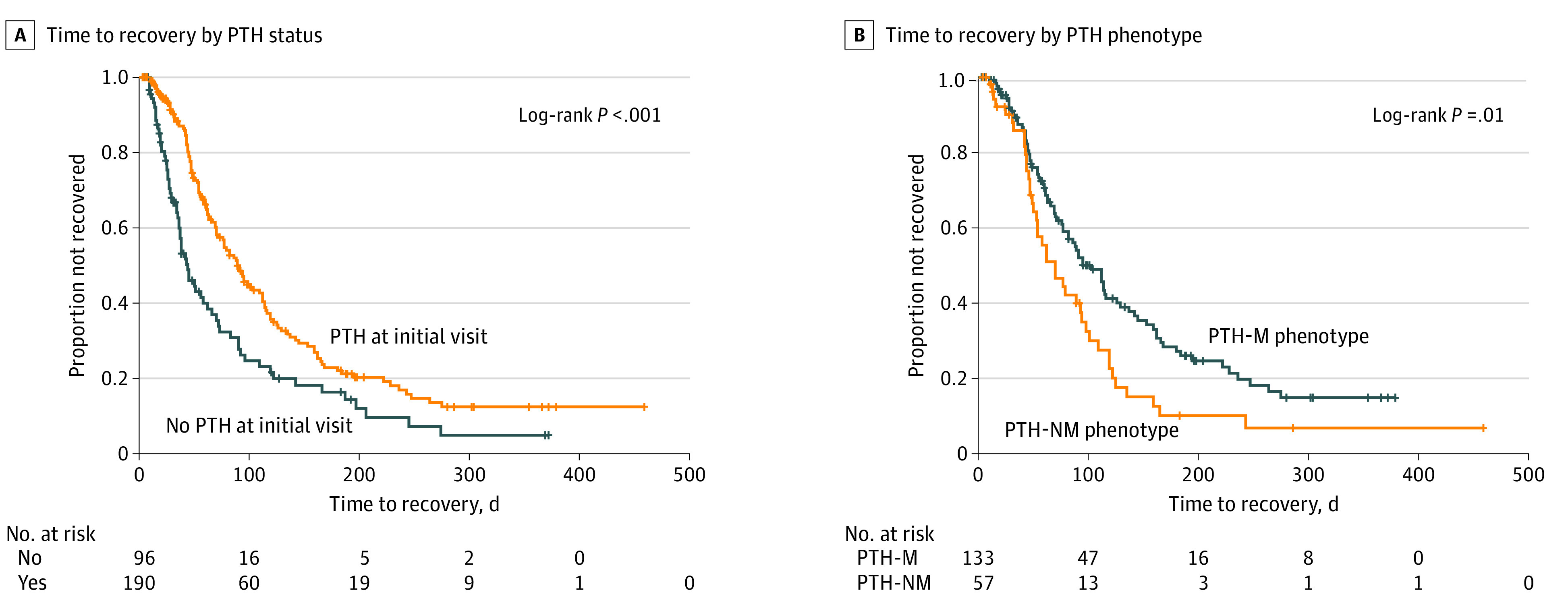
Kaplan-Meier Curve for Posttraumatic Headache Recovery by Status PTH-M indicates posttraumatic headache experienced by a patient with a migraine phenotype; PTH-NM, headache experienced by a patient without a migraine phenotype.

**Figure 2.**
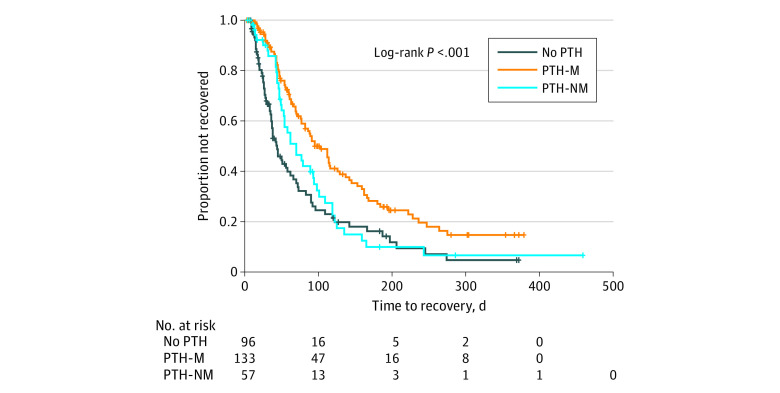
Kaplan-Meier Curve for Time to Recovery by Posttraumatic Headache Status PTH-M indicates posttraumatic headache experienced by a patient with a migraine phenotype; PTH-NM, headache experienced by a patient without a migraine phenotype.

Patients with any type of PTH after concussion were more likely to have prolonged recovery than those without PTH (log-rank *P* < .001) (Figure 1). Patients in the PTH-M group took significantly longer to recover than the PTH-NM group (median [interquartile range], 95 [54-195] days vs 70 [46-119]; log-rank *P* = .01) (Figure 1). The global log-rank test confirmed there were differences between groups (Figure 2). While the acute postconcussion recovery rate appears slower for PTH-NM than the no PTH group, the recovery trajectories of these 2 groups begin to overlap around 100 days; however, both recovery rates are faster than for youth with PTH-M throughout the study period.

PTH 3 months and 6 months following injury was more common in those with PTH-M than PTH-NM, but this difference was only significant in the univariable analysis at 3 months (Table 2). Given the strong association between female sex and prolonged recovery in prior studies,^1,2,17^ recovery time and PTH at 3 months was analyzed in each PTH group to compare the sexes. Within each PTH phenotype, there was no significant difference in recovery or rates of PTH at 3 months between the sexes (eTable 5 in the Supplement).

**Table 2.** Outcome Characteristics by Headache Status at Initial Clinic Visit

Outcome	Concussions, No. (%)^a^				*P* value for PTH-M vs PTH-NM
	Headache phenotype at initial visit			Overall (n = 286)	
	PTH-M (n = 133)	PTH-NM (n = 57)	No PTH (n = 96)		
Headache, follow-up					
At 3 mo	27/92 (29.3)	5/41 (12.2)	8/70 (11.4)	40/203 (19.7)	.05^b^
At 6 mo	10/93 (10.8)	2/44 (4.5)	4/71 (5.6)	16/208 (7.7)	.34^b^
Time to recovery					
Concussions tracked by median, No.^d^	83	41	67	191	.01^c^
Median (IQR), d	95 (54-195)	70 (46-119)	44 (26-96)	72 (42-159)	

Abbreviations: IQR, interquartile range; PTH-M, posttraumatic headache with migraine; PTH-NM, posttraumatic headache with nonmigraine.

^a^ Patients may be enrolled for more than 1 concussion at different time points; data summarized is concussion level.

^b^ Determined with a Fisher exact test.

^c^ Determined with a log-rank test.

^d^ Used recovery date from clinician for 31/83 (37%), 19/41 (46%), 27/67 (40%), and 77/191 (40%), respectively.

As noted in the statistical analysis section, the multivariable models were intended to contain phenotype × sex interactions. However, model estimates were unwieldy with the interactions and none of the adjusted effects were statistically significant. The decision was made to remove the interaction terms, whereupon the adjusted associations were more stably estimated. The multivariable models indicate that the association between initial PTH phenotype and both recovery time and PTH at 3 months after injury was significant while adjusting for age and sex (time to recovery: hazard ratio, 0.64; 95% CI, 0.43-0.93; *P* = .02; headache at 3 months: odds ratio, 3.08; 95% CI, 1.14-9.92; *P* = .02). Neither age nor sex were significantly related to time to recovery or concussion-attributable headache at 3 months in the multivariable model (Table 3).

**Table 3.** Multivariable Models of Factors Associated With Time to Recovery and Headache 3 Months After Injury^a^

Characteristic	Time to recovery, d^b^		Headache at 3 mo postinjury^c^	
	Hazard ratio (95% CI)	*P* value	Odds ratio (95% CI)	*P* value
PTH phenotype				
Nonmigraine	1 [Reference]	.02	1 [Reference]	.02
Migraine	0.64 (0.43-0.93)		3.08 (1.14-9.92)	
Age at injury, y				
5-12	1 [Reference]	.38	1 [Reference]	.76
13-18	0.77 (0.42-1.40)		1.23 (0.34-5.82)	
Sex				
Male	1 [Reference]	.63	1 [Reference]	.80
Female	0.91 (0.62-1.34)		0.89 (0.37-2.23)	

^a^ Patients may be enrolled for more than 1 concussion at different time points; data summarized is concussion level.

^b^ Results for time to recovery are based on a Cox proportional hazards multivariable model, adjusting for each of the predictors in this table.

^c^ Results for headache at 3 months are based on a logistic regression multivariable model, adjusting for each of the predictors in this table.

## Discussion

This cohort study is the first, to our knowledge, to collect data regarding PTH phenotypes in youth and analyze the association between PTH phenotypes and concussion treatment and recovery. We found PTH with migraine phenotype was associated with persistent symptoms following concussion compared with no headache or nonmigraine PTH. Thus, while PTH is one of the most persistent and challenging symptoms following concussion, headache itself does not appear to indicate a single trajectory; it is the headache phenotype that correlates with outcome. Therefore, phenotyping PTH may convey important prognostic information, may guide treatment, and could be essential for successful clinical treatment trials. In addition, detailed characterization of headache after concussion may advance our understanding of PTH.

Although the mechanism of PTH is not fully understood, it has been hypothesized that concussion and migraine may have similar underlying processes. This could explain why the majority of PTH have migraine qualities,^18^ and why a history of migraine has been associated with prolonged recovery after concussion.^2,19^ While the pathophysiology of concussion and migraine is complex, many physiological changes following concussion are similar to those described in migraine, including ionic flux, disruption to cerebral metabolism and alteration of neurotransmitters and neuropeptides such as glutamate, calcitonin gene-related peptide, pituitary adenylate-cyclase-activating polypeptide, and substance P.^19^

It is striking that there are few differences in patient characteristics between the PTH-M and PTH-NM groups, indicating that migraine phenotype itself may be a marker of risk for prolonged recovery following concussion. A migraine phenotype also offers a target for early intervention, as there are evidence-based treatments for migraine that could potentially improve outcome and manage headache. This may also suggest potential for objective biomarkers (eg, molecular, imaging) to help distinguish between different PTH phenotypes.

In addition, within each headache phenotype there was no significant sex-related difference in time to recovery nor risk of concussion-related headache 3 months after injury. Prior studies that did not describe PTH phenotype have found that girls have a higher risk for PTH and for prolonged recovery after concussion than boys.^2,17,20^ Our results suggest it may be the migraine PTH phenotype, not simply female sex, which is associated with prolonged recovery. If true, girls may not have a higher risk of persistent symptoms than boys with a similar headache phenotype, but rather a higher risk for migraine PTH.

Given that time to clinical presentation was similar between PTH-M and PTH-NM groups, differences in recovery were unrelated to time from injury.^21,22^ The finding that personal history of migraine is 10-fold more common in those with PTH-M than PTH-NM, and that headaches at baseline were more common in the PTH-M group, has been described in adult PTH studies and suggests that mTBI may be triggering an underlying risk for migraine in genetically susceptible individuals.^23^

As there are few randomized trials of pharmacological interventions to treat concussion symptoms or PTH, this study’s description of treatments is particularly relevant. A retrospective study of youth with PTH found that melatonin was as effective as prescription preventive medications, which may explain the recommendation of melatonin to 30% of participants.^18^ Unfortunately, a 2020 randomized clinical trial of melatonin^24^ did not support its use for postconcussive symptoms. It is interesting that nutraceuticals were recommended to over 37% of both groups while preventive medications were not commonly prescribed. This is consistent with a survey of pediatric PTH treatment that found most clinicians would wait 4 weeks or more after injury to start preventive medications.^25^ Both the survey and this study found that the most popular prescription medications were tricyclic antidepressants, despite the facts that amitriptyline was not superior to placebo for pediatric migraine prevention^9^ and may cause significant adverse effects. This study provides information about which treatments are being used for PTH, but use of medications or nutraceuticals is not data driven as there are few prospective studies of these treatments to manage PTH. This is a critical knowledge gap that needs to be addressed.

### Limitations

There are several limitations to this study. The definition of migraine headache using PCSI data should be further validated. However, this definition was more selective than prior studies of PTH, and criteria from *ICHD-3* was included in this definition.^5,7^ In addition, other PTH studies have found approximately 50% of such headaches meet migraine criteria.^18,23^ While we found 69% of PTH were PTH-M, the proportion of youth with severe symptoms is likely high in this cohort recruited from specialty concussion clinics. We also did not differentiate between a completely new headache after concussion and those with a primary headache syndrome exacerbated by concussion because the *ICHD-3* definition of PTH does not make this distinction.^5^ Future studies will be strengthened by prospective classification of PTH phenotype using validated criteria.

There were some differences between patients who were excluded and included in the initial study population, which could have introduced bias. There were higher proportions of boys, younger children, patients using public insurance, and patients with a family history of ADHD in the excluded group than the study cohort. Female sex and adolescent age have been associated with prolonged recovery after concussion,^2^ so the study cohort might have had a higher risk of prolonged recovery than the excluded group. However, the excluded group had a great deal of missing data, making accurate comparisons difficult. The inclusion of 2 concussions for each of 5 patients as though from 10 unique patients has negligible statistical impact and was not sufficiently common to require repeated-measures analysis.

It is possible that those with PTH-M simply had more symptoms than those with PTH-NM, and higher symptom count following concussion has been associated with prolonged recovery.^1,2^ However, only 4 of 21 symptoms on the PCSI were used to define PTH-M, so high symptom count alone is unlikely to be responsible for the association between PTH-M and prolonged recovery. We also did not have data about disability related to headache, and as with all scales, a certain rating on the PCSI may mean different things to each patient.

Follow-up data were not available for all patients at 3 and 6 months owing to incomplete data and patients lost to follow-up. While the follow-up data may not reflect findings from the whole group because of potential bias, a similar percentage of each group are included in the baseline and follow-up data. We assumed that those who recovered before follow-up survey completion did not have PTH after recovery, which may underestimate the true number of patients with PTH at follow-up. There may also be bias in recall, as patients with concussion may be more likely to believe they had fewer or less severe headaches in the past, as per the “good-old days” bias, which could result in an overestimation of the number of patients with true worsening of headache following concussion.^26^ In addition, there may be bias due to the differences in the method of reporting recovery—some participants had recovery reported by a medical professional based on an in-person clinic visit and others had recovery reported by parental survey—although the proportions of recovery determined in each fashion was similar between groups.

There is an inherent limitation to clinical registry patients that have been referred to specialty TBI clinics. These patients may have higher symptom burdens and prolonged recovery, and therefore could have a higher prevalence of previously described risk factors such as adolescent age and female sex. This could skew data to a more prolonged recovery and decrease generalizability to nonspecialty populations.

## Conclusions

In this study, PTH with migraine phenotype in the acute period following concussion was common among those seeking care after concussion and was associated with prolonged recovery compared with nonmigraine PTH or no PTH. Risk of concussion-related headaches 3 months after injury was also higher for those with PTH-M compared with PTH-NM. The presence of PTH-M following concussion may be a more important risk factor for prolonged recovery than any PTH or sex. Female sex has been a consistent, but incompletely understood, risk for prolonged recovery following concussion.^2^ This study suggests that PTH phenotype may begin to explain this finding. While migraine PTH phenotype was more common in girls, there was no significant difference in recovery time between the sexes within each PTH phenotype (ie, no difference in recovery time between boys and girls who share the same PTH phenotype) and there was no difference in recovery time between the sexes after controlling for PTH phenotype in multivariable analysis. Thus, PTH with a migraine phenotype may indicate a more deleterious pathophysiology following concussion, which leads to a higher risk of persistent symptoms and could be a target for early intervention following concussion.

Future large studies validating the classification of posttraumatic headache phenotypes in youth and studying outcomes are essential. PTH phenotyping will improve prognostication of concussion recovery and will enhance the treatment for PTH with more appropriate and targeted therapies to treat and prevent persistent and disabling headaches in youth with concussion.

## Notes

Supplement. **eTable 1. **Comparison of Patient Characteristics, Included vs. Excluded Subjects **eTable 2. **Non-Significant Patient and Event Characteristics by Headache Status at Initial Clinic Visit **eTable 3. **Treatment Provided at Initial Clinic Visit **eTable 4. **Medications and Nutraceuticals Recommended for PTH, Acute and Preventive Treatments **eTable 5. **Outcome for Headache Phenotype by Sex **eFigure. **Flow Chart of Included vs. Excluded Subjects
