# Supplementary material for: Evaluation of Posttraumatic Headache Phenotype and Recovery Time After Youth Concussion
Source: JAMA Netw Open. 2021 Mar 8;4(3):e211312. doi: 10.1001/jamanetworkopen.2021.1312 (PMC7941198; doi:10.1001/jamanetworkopen.2021.1312)
Supplement: Supplement. — eTable 1. Comparison of Patient Characteristics, Included vs. Excluded Subjects eTable 2. Non-Significant Patient and Event Characteristics by Headache Status at Initial Clinic Visit eTable 3. Treatment Provided at Initial Clinic Visit eTable 4. Medications and Nutraceuticals Recommended for PTH, Acute and Preventive Treatments eTable 5. Outcome for Headache Phenotype by Sex eFigure. Flow Chart of Included vs. Excluded Subjects [file jamanetwopen-e211312-s001.pdf]

Supplemental Online Content

Kamins J, Richards R, Barney BJ, et al. Evaluation of posttraumatic headache phenotype and recovery time after youth concussion. *JAMA Netw Open*. 2021;4(3):e211312. doi:10.1001/jamanetworkopen.2021.1312

eTable 1. Comparison of Patient Characteristics, Included vs. Excluded Subjects

eTable 2. Non-Significant Patient and Event Characteristics by Headache Status at Initial Clinic Visit

eTable 3. Treatment Provided at Initial Clinic Visit

eTable 4. Medications and Nutraceuticals Recommended for PTH, Acute and Preventive Treatments

eTable 5. Outcome for Headache Phenotype by Sex

eFigure. Flow Chart of Included vs. Excluded Subjects

This supplemental material has been provided by the authors to give readers additional information about their work.

© 2021 Kamins J et al. *JAMA Network Open.*

eTable 1. Comparison of Patient Characteristics, Included vs. Excluded Subjects

	Included		
	No (N = 339)	Yes (N = 286)	P-value
**Demographics**			
**Age at injury**			**<.001[a]**
5 - 12 years old	123/327 (37.6%)	48/286 (16.8%)	
13 - 18 years old	204/327 (62.4%)	238/286 (83.2%)	
**Sex**			**.03[a]**
Male	165/328 (50.3%)	118/286 (41.3%)	
Female	163/328 (49.7%)	168/286 (58.7%)	
**Ethnicity**			.09[a]
Not Hispanic or Latino	256/284 (90.1%)	228/268 (85.1%)	
Hispanic or Latino	28/284 (9.9%)	40/268 (14.9%)	
**Race**			.50[a]
American Indian/Native Hawaiian	3/250 (1.2%)	4/258 (1.6%)	
Asian	18/250 (7.2%)	10/258 (3.9%)	
African American	24/250 (9.6%)	23/258 (8.9%)	
White	185/250 (74.0%)	195/258 (75.6%)	
Multiracial	20/250 (8.0%)	26/258 (10.1%)	
**Insurance type**			**.005[a]**
Medicaid/State Child Health Insurance Plan (CHIP)	63/326 (19.3%)	28/284 (9.9%)	
Commercial Insurance	257/326 (78.8%)	249/284 (87.7%)	
Medicare	1/326 (0.3%)	1/284 (0.4%)	
No Insurance/Self Pay	5/326 (1.5%)	6/284 (2.1%)	
**Highest parent education level**			.37[a]
Some high school or less	1/100 (1.0%)	11/281 (3.9%)	
High school graduate or GED	5/100 (5.0%)	11/281 (3.9%)	
Vocational school or some college	7/100 (7.0%)	32/281 (11.4%)	
College degree	40/100 (40.0%)	94/281 (33.5%)	
Master's or doctoral degree	47/100 (47.0%)	133/281 (47.3%)	
**Educational services received prior to concussion**			.10[a]
Special Education (IEP, 504 Plan)	61/299 (20.4%)	41/272 (15.1%)	
Regular Education	238/299 (79.6%)	231/272 (84.9%)	
**Patient Medical History**			
ADHD	47/296 (15.9%)	38/261 (14.6%)	.72[a]
Anxiety	65/300 (21.7%)	63/268 (23.5%)	.62[a]
Depression	32/293 (10.9%)	26/262 (9.9%)	.78[a]

© 2021 Kamins J et al. *JAMA Network Open.*

eTable 1. Comparison of Patient Characteristics, Included vs. Excluded Subjects

	Included		
	No (N = 339)	Yes (N = 286)	P-value
Learning Disabilities	28/290 (9.7%)	23/261 (8.8%)	.77[a]
Sleep Problems	29/291 (10.0%)	31/260 (11.9%)	.50[a]
Seizures	5/287 (1.7%)	7/259 (2.7%)	.56[a]
Migraines	47/293 (16.0%)	55/259 (21.2%)	.12[a]
Prior concussions	169/332 (50.9%)	134/280 (47.9%)	.47[a]
Total number of comorbidities (excluding seizures)			.09[b]
0	84/261 (32.2%)	59/230 (25.7%)	
1	108/261 (41.4%)	96/230 (41.7%)	
2	33/261 (12.6%)	40/230 (17.4%)	
3 or more	36/261 (13.8%)	35/230 (15.2%)	
**Family Medical History**			
Migraines	99/262 (37.8%)	113/248 (45.6%)	.09[a]
Depression	94/262 (35.9%)	66/229 (28.8%)	.10[a]
Anxiety	98/263 (37.3%)	75/227 (33.0%)	.34[a]
ADHD	71/255 (27.8%)	40/223 (17.9%)	.01[a]
Learning Disabilities	49/253 (19.4%)	34/226 (15.0%)	.23[a]

[a]Fisher's exact test.
[b]Kruskal-Wallis test.
Patients may be enrolled for more than one concussion at different time points; data summarized is concussion level.

© 2021 Kamins J et al. *JAMA Network Open.*

eTable 2. Non-Significant Patient and Event Characteristics by Headache Status at Initial Clinic Visit

	Headache phenotype at initial visit				
	PTH Migraine (N = 133)	PTH Non-Migraine (N = 57)	No PTH (N = 96)	Overall (N = 286)	P-value for PTH-M vs. PTH-NM
**Demographics**					
**Ethnicity**					.81[a]
Not Hispanic or Latino	109/127 (85.8%)	47/53 (88.7%)	72/88 (81.8%)	228/268 (85.1%)	
Hispanic or Latino	18/127 (14.2%)	6/53 (11.3%)	16/88 (18.2%)	40/268 (14.9%)	
**Race**					.43[a]
American Indian/Native Hawaiian	0/120 (0.0%)	0/53 (0.0%)	4/85 (4.7%)	4/258 (1.6%)	
Asian	2/120 (1.7%)	3/53 (5.7%)	5/85 (5.9%)	10/258 (3.9%)	
African American	11/120 (9.2%)	3/53 (5.7%)	9/85 (10.6%)	23/258 (8.9%)	
White	96/120 (80.0%)	41/53 (77.4%)	58/85 (68.2%)	195/258 (75.6%)	
Multiracial	11/120 (9.2%)	6/53 (11.3%)	9/85 (10.6%)	26/258 (10.1%)	
**Highest parent education level**					.16[a]
Some high school or less	5/129 (3.9%)	3/57 (5.3%)	3/95 (3.2%)	11/281 (3.9%)	
High school graduate or GED	7/129 (5.4%)	1/57 (1.8%)	3/95 (3.2%)	11/281 (3.9%)	
Vocational school or some college	21/129 (16.3%)	3/57 (5.3%)	8/95 (8.4%)	32/281 (11.4%)	
College degree	38/129 (29.5%)	22/57 (38.6%)	34/95 (35.8%)	94/281 (33.5%)	
Master's or doctoral degree	58/129 (45.0%)	28/57 (49.1%)	47/95 (49.5%)	133/281 (47.3%)	
**Educational services received prior to concussion**					.67[a]
Special Education (IEP, 504 Plan)	20/126 (15.9%)	10/54 (18.5%)	11/92 (12.0%)	41/272 (15.1%)	
Regular Education	106/126 (84.1%)	44/54 (81.5%)	81/92 (88.0%)	231/272 (84.9%)	
**Patient Medical History**					
**ADHD**	18/120 (15.0%)	11/53 (20.8%)	9/88 (10.2%)	38/261 (14.6%)	.38[a]
**Anxiety**	33/123 (26.8%)	9/54 (16.7%)	21/91 (23.1%)	63/268 (23.5%)	.18[a]
**Learning Disabilities**	10/119 (8.4%)	5/53 (9.4%)	8/89 (9.0%)	23/261 (8.8%)	.78[a]
**Sleep Problems**	17/121 (14.0%)	3/52 (5.8%)	11/87 (12.6%)	31/260 (11.9%)	.19[a]
**Seizures**	5/121 (4.1%)	0/52 (0.0%)	2/86 (2.3%)	7/259 (2.7%)	.32[a]
**Prior concussions**	61/130 (46.9%)	25/56 (44.6%)	48/94 (51.1%)	134/280 (47.9%)	.87[a]
**Total number of comorbidities (excluding seizures)**					.12[b]
0	23/106 (21.7%)	13/45 (28.9%)	23/79 (29.1%)	59/230 (25.7%)	
1	48/106 (45.3%)	22/45 (48.9%)	26/79 (32.9%)	96/230 (41.7%)	

© 2021 Kamins J et al. *JAMA Network Open.*

eTable 2. Non-Significant Patient and Event Characteristics by Headache Status at Initial Clinic Visit

	Headache phenotype at initial visit				
	PTH Migraine (N = 133)	PTH Non-Migraine (N = 57)	No PTH (N = 96)	Overall (N = 286)	P-value for PTH-M vs. PTH-NM
2	17/106 (16.0%)	7/45 (15.6%)	16/79 (20.3%)	40/230 (17.4%)	
3 or more	18/106 (17.0%)	3/45 (6.7%)	14/79 (17.7%)	35/230 (15.2%)	
**Family Medical History**					
**Migraines**	57/112 (50.9%)	21/49 (42.9%)	35/87 (40.2%)	113/248 (45.6%)	.39[a]
**Depression**	34/102 (33.3%)	11/46 (23.9%)	21/81 (25.9%)	66/229 (28.8%)	.33[a]
**Anxiety**	32/100 (32.0%)	14/47 (29.8%)	29/80 (36.3%)	75/227 (33.0%)	.85[a]
**Learning Disabilities**	16/99 (16.2%)	3/46 (6.5%)	15/81 (18.5%)	34/226 (15.0%)	.12[a]
**Event Characteristics**					
**Time to initial clinic visit (days)**					.74[b]
N	133	57	96	286	
median [q1,q3]	18.0 [9.0,30.0]	19.0 [7.0,27.0]	17.0 [10.0,29.0]	18.0 [9.0,29.0]	
**Onset of typical symptoms within 24-48 hours of the injury event**					.45[a]
Yes (typical symptoms AND onset < 24-48 hours)	125/131 (95.4%)	50/55 (90.9%)	86/95 (90.5%)	261/281 (92.9%)	
No (atypical symptoms)	3/131 (2.3%)	2/55 (3.6%)	6/95 (6.3%)	11/281 (3.9%)	
No (delayed onset)	3/131 (2.3%)	3/55 (5.5%)	3/95 (3.2%)	9/281 (3.2%)	
**Gradual recovery of symptoms over the first week of the injury**					.17[a]
No (There have been worsening symptoms over the first week)	18/130 (13.8%)	3/55 (5.5%)	7/94 (7.4%)	28/279 (10.0%)	
Yes (There have been improving symptoms over the first week)	58/130 (44.6%)	31/55 (56.4%)	77/94 (81.9%)	166/279 (59.5%)	
Yes (There have been stable symptoms over the first week)	54/130 (41.5%)	21/55 (38.2%)	10/94 (10.6%)	85/279 (30.5%)	
**Definite reported mechanism of injury**					>.99[a]
Yes (There was a discrete event with force to the head or rapid head movement without impact)	128/131 (97.7%)	54/55 (98.2%)	93/95 (97.9%)	275/281 (97.9%)	
No (There was no discrete event)	3/131 (2.3%)	1/55 (1.8%)	2/95 (2.1%)	6/281 (2.1%)	
**Mechanism/cause of injury**					.45[a]
Motor vehicle crash (MVC)	9/130 (6.9%)	3/56 (5.4%)	2/94 (2.1%)	14/280 (5.0%)	
Assault	6/130 (4.6%)	1/56 (1.8%)	1/94 (1.1%)	8/280 (2.9%)	
Struck by or against object/person (non-assault, non-sport)	16/130 (12.3%)	3/56 (5.4%)	8/94 (8.5%)	27/280 (9.6%)	
Fall (non-sport)	12/130 (9.2%)	8/56 (14.3%)	5/94 (5.3%)	25/280 (8.9%)	

© 2021 Kamins J et al. *JAMA Network Open.*

eTable 2. Non-Significant Patient and Event Characteristics by Headache Status at Initial Clinic Visit

	Headache phenotype at initial visit				
	PTH Migraine (N = 133)	PTH Non-Migraine (N = 57)	No PTH (N = 96)	Overall (N = 286)	P-value for PTH-M vs. PTH-NM
Sport/Recreation	87/130 (66.9%)	41/56 (73.2%)	78/94 (83.0%)	206/280 (73.6%)	
**Retrograde Amnesia**	17/124 (13.7%)	6/55 (10.9%)	8/90 (8.9%)	31/269 (11.5%)	.81[a]
**Anterograde Amnesia**	20/123 (16.3%)	11/55 (20.0%)	16/90 (17.8%)	47/268 (17.5%)	.53[a]
**Loss of consciousness**[c]	7/123 (5.7%)	5/53 (9.4%)	15/87 (17.2%)	27/263 (10.3%)	.35[a]

[a]Fisher's exact test.
[b]Kruskal-Wallis test.
[c]An exploratory Fisher's exact test for LOC between new headache and no headache p=.02.
Patients may be enrolled for more than one concussion at different time points; data summarized is concussion level.

© 2021 Kamins J et al. *JAMA Network Open.*

eTable 3. Treatment Provided at Initial Clinic Visit

	PTH Phenotype			
	PTH Migraine (N = 133)	PTH Non-Migraine (N = 57)	Overall (N = 190)	P-value
**Cognitive Treatment**				
General education/counseling regarding management of cognitive load/activity related to symptom increase	122 (91.7%)	51 (89.5%)	173 (91.1%)	.59
Taught strategies for improving attention, memory, and/or organization	3 (2.3%)	1 (1.8%)	4 (2.1%)	>.99
Over the counter medication/supplement	1 (0.8%)	0 (0.0%)	1 (0.5%)	>.99
Recommendation regarding level of cognitive activity	118 (88.7%)	42 (73.7%)	160 (84.2%)	.02
**Physical Treatment**				
General education/counseling regarding appropriate level of tolerable physical activity, its benefits/risks	123 (92.5%)	52 (91.2%)	175 (92.1%)	.77
Rehabilitation/ therapy: e.g. aerobic, physical conditioning	7 (5.3%)	1 (1.8%)	8 (4.2%)	.44
Vestibular exercises/treatment	0 (0.0%)	1 (1.8%)	1 (0.5%)	.30
Cervical spinal/musculoskeletal	2 (1.5%)	0 (0.0%)	2 (1.1%)	>.99
Recommendation regarding level of physical activity	123 (92.5%)	50 (87.7%)	173 (91.1%)	.28
**Headache Treatment**				
General education/counseling	127 (95.5%)	50 (87.7%)	177 (93.2%)	.06
Directed strategies, including headache diary, recommendations about diet/ hydration	36 (27.1%)	13 (22.8%)	49 (25.8%)	.59
Over the counter medication/supplement	59 (44.4%)	24 (42.1%)	83 (43.7%)	.87
Prescription medication	30 (22.6%)	6 (10.5%)	36 (18.9%)	.07
**Sleep-Related Treatment**				
General education/ counseling regarding good sleep hygiene/ habits	116 (87.2%)	49 (86.0%)	165 (86.8%)	.82
Directed strategies for sleep hygiene (e.g., scheduling, strategies for insomnia)	20 (15.0%)	7 (12.3%)	27 (14.2%)	.82
Over the counter medication/supplement	34 (25.6%)	12 (21.1%)	46 (24.2%)	.58
Prescription medication	1 (0.8%)	0 (0.0%)	1 (0.5%)	>.99
**Emotion-Related Treatment**				

© 2021 Kamins J et al. *JAMA Network Open.*

eTable 3. Treatment Provided at Initial Clinic Visit

	PTH Phenotype			
	PTH Migraine (N = 133)	PTH Non-Migraine (N = 57)	Overall (N = 190)	P-value
General education regarding emotional response to injury (e.g. frustration, irritability, anxiety, depressed mood) and to activity restrictions/ limitations	112 (84.2%)	46 (80.7%)	158 (83.2%)	.53
Problem-specific treatments: (e.g. therapy for anxiety, depressed mood)	7 (5.3%)	3 (5.3%)	10 (5.3%)	>.99
Prescription medication	4 (3.0%)	0 (0.0%)	4 (2.1%)	.32

Fisher's exact test was used for all comparisons.

Patients may be enrolled for more than one concussion at different time points; data summarized is concussion level.

© 2021 Kamins J et al. *JAMA Network Open.*

eTable 4. Medications and Nutraceuticals Recommended for PTH, Acute and Preventive Treatments

	Initial Clinic Visit			Any Clinic Visit		
	Migraine PTH (N = 133)	Non-Migraine PTH (N = 57)	P-value	Migraine PTH (N = 133)	Non-Migraine PTH (N = 57)	P-value
**Abortive Medications**						
Any abortive medication(s)	61 (45.9%)	28 (49.1%)		62 (46.6%)	28 (49.1%)	
Acetaminophen/Tylenol	53 (39.8%)	22 (38.6%)		53 (39.8%)	23 (40.4%)	
Ibuprofen/Advil/Motrin	50 (37.6%)	24 (42.1%)		52 (39.1%)	24 (42.1%)	
Naproxen	11 (8.3%)	9 (15.8%)		12 (9.0%)	9 (15.8%)	
Steroid (other)	1 (0.8%)	1 (1.8%)		1 (0.8%)	1 (1.8%)	
Rizatriptan/Maxalt	4 (3.0%)	0 (0.0%)		6 (4.5%)	0 (0.0%)	
Zolmitriptan/Zomig				1 (0.8%)	1 (1.8%)	
Sumatriptan/Imitrex	1 (0.8%)	1 (1.8%)		1 (0.8%)	1 (1.8%)	
**Preventive Medications**						
Any preventive medication(s)	24 (18.0%)	4 (7.0%)	.05[a]	28 (21.1%)	5 (8.8%)	.04[a]
Propranolol/Inderal	1 (0.8%)	0 (0.0%)		2 (1.5%)	0 (0.0%)	
Cyproheptadine	1 (0.8%)	0 (0.0%)		1 (0.8%)	0 (0.0%)	
Gabapentin/Neurontin	3 (2.3%)	0 (0.0%)		3 (2.3%)	0 (0.0%)	
Memantine/Namenda	2 (1.5%)	0 (0.0%)		4 (3.0%)	0 (0.0%)	
Venlafaxine/Effexor	1 (0.8%)	0 (0.0%)		1 (0.8%)	0 (0.0%)	
Duloxetine/Cymbalta	3 (2.3%)	1 (1.8%)		3 (2.3%)	2 (3.5%)	
Topiramate/ Topamax	1 (0.8%)	0 (0.0%)		1 (0.8%)	0 (0.0%)	
Amitriptyline/Elavil	10 (7.5%)	1 (1.8%)		11 (8.3%)	1 (1.8%)	
Nortriptyline/Pamelor	5 (3.8%)	2 (3.5%)		5 (3.8%)	2 (3.5%)	
**Nutraceuticals**						
Any nutraceutical(s)	50 (37.6%)	20 (35.1%)		53 (39.8%)	21 (36.8%)	
B2/Riboflavin	27 (20.3%)	12 (21.1%)		33 (24.8%)	13 (22.8%)	
Butterbur	11 (8.3%)	5 (8.8%)		11 (8.3%)	5 (8.8%)	
Magnesium	30 (22.6%)	14 (24.6%)		36 (27.1%)	15 (26.3%)	
Melatonin	40 (30.1%)	13 (22.8%)		41 (30.8%)	14 (24.6%)	

[a]Chi-square p-values are calculated based on patient receiving preventive medication prescription, regardless of how many preventive medications were prescribed. These p-values are from data-driven tests, not pre-specified.

Patients may be enrolled for more than one concussion at different time points; data summarized is concussion level.

© 2021 Kamins J et al. *JAMA Network Open.*

eTable 5: Outcome for Headache Phenotype by Sex

Headache Status	Time to Concussion Symptom Resolution (days)[a]<br>N, Median[b] [Q1, Q3]	Symptom Resolution p-value	Percent with Headache at 3 months[c]<br>n/N (%)	Headache p-value
**PTH Migraine**		.69[d]		.79[e]
Male	21<br>**95** [43, 162]		6/24 (25.0%)	
Female	62<br>**103** [56, 222]		21/68 (30.9%)	
All	83<br>**95** [54, 195]		27/92 (29.3%)	
**PTH Non-Migraine**		.79[d]		.18[e]
Male	19<br>**58** [46, 119]		4/20 (20.0%)	
Female	22<br>**77** [44, 109]		1/21 (4.8%)	
All	41<br>**70** [46, 119]		5/41 (12.2%)	
**No PTH**		.46[d]		>.99[e]
Male	38<br>**38** [25, 119]		4/39 (10.3%)	
Female	29<br>**45** [26, 96]		4/31 (12.9%)	
All	67<br>**44** [26, 96]		8/70 (11.4%)	

[a]Defined as (recovery date - injury date).
[b]Median and IQR were calculated accounting for censoring.
[c]Percentages are based on the total number of subjects in each group who either completed a 3-month follow-up survey or recovered within 104 days.
[d]Logrank test.
[e]Fisher's exact test.
Patients may be enrolled for more than one concussion at different time points; data summarized is concussion level.

© 2021 Kamins J et al. *JAMA Network Open.*

eFigure. Flow Chart of Included vs. Excluded Subjects

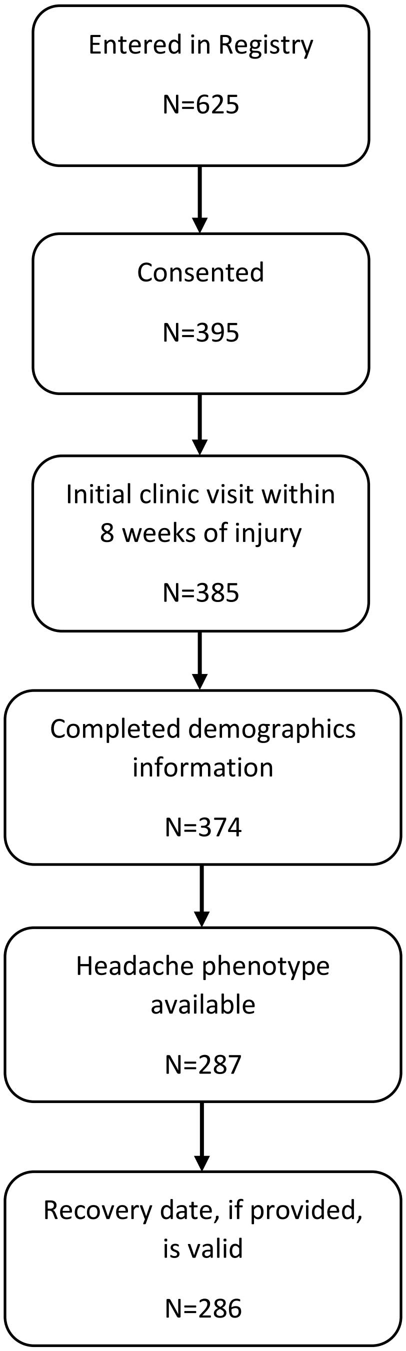

Patients may be enrolled for more than one concussion at different time points; depicted flow is concussion level.

© 2021 Kamins J et al. *JAMA Network Open.*
